# Clinical Pathway in the Treatment of Nocardial Brain Abscesses following Systemic Infections

**DOI:** 10.1155/2014/584934

**Published:** 2014-08-28

**Authors:** Yun-Cong Zheng, Tse-Lun Wang, Jee-Ching Hsu, Yung-Hsing Hsu, Wen-Hsing Hsu, Chih-Liang Wang, Aij-Lie Kwan, Chih-Lung Lin

**Affiliations:** ^1^Department of Neurosurgery, Chang Gung Memorial Hospital and University, Taoyuan, Taiwan; ^2^Department of Neurosurgery, Kaohsiung Medical University, Kaohsiung, Taiwan; ^3^Department of Anesthesiology, Chang Gung Memorial Hospital and University, Taoyuan, Taiwan; ^4^Department of Chest, Chang Gung Memorial Hospital and University, Taoyuan, Taiwan

## Abstract

Nocardial infections are commonly encountered in patients with immunocompromised states. Cerebral nocardiosis is an uncommon clinical entity, representing only 2% of all cerebral abscesses. It has a higher mortality rate, especially for multiple cerebral lesions in immunocompromised hosts following systemic infections. However, an optimal treatment policy to deal with these immunocompromised patients in Asia is still lacking. We retrospectively reviewed the subjects with nocardial brain abscesses from 2001 to 2011 at our medical center. All of them had multiple brain abscesses, underlying with immunocompromised state following systemic infections. All cases were under steroid control due to their comorbidities for more than six months. The comorbidities and misdiagnosis often lead to poor prognosis. The change in the environments of the microorganisms caused by immunosuppressive agents and multiple antibiotic uses may play an important role in this critical disorder. Aggressive craniotomy should be performed in time to avoid grievous neurological outcomes. Our conclusion is that early diagnosis and appropriate antibiotic uses should be implemented promptly, and aggressive craniotomy should be performed for nocardial brain abscesses in subjects with systemic infections under an immunocompromised status.

## 1. Introduction

Cerebral nocardiosis is an uncommon clinical entity, representing only 2% of all cerebral abscesses [1, 2]. Current literature exists in the form of anecdotal reports, small case series, and retrospective studies, lacking prospective studies. An optimal treatment protocol for the management of nocardial brain abscesses has not been established yet. Few of the associated articles reported deal with cases in East Asia. We will share our experiences at our institute in Taiwan and compare it to related articles to develop a treatment policy that includes operation and medical methods to manage nocardial brain abscesses in immunocompromised patients.

## 2. Patients and Methods

We retrospectively traced the patients with nocardial brain abscesses from 2001 to 2011 at our institute. The information about the character of the abscesses, location, symptoms/signs, treatments, and clinical outcomes was obtained by reviewing the charts and radiological reports. Three patients were identified according to the laboratory cultures based on the bacteria species obtained from the cerebral abscesses. Of these three patients, one was male and two were female. All of them had an immunocompromised status and concurrent steroid use. The clinical course, radiological findings, and the management of the nocardial abscesses in these cases are summarized in Table 1.

### 2.1. Case 1

A 40-year-old housewife with systemic sclerosis with Raynaud's syndrome was diagnosed with pulmonary tuberculosis in 1999 and reported having herpes zoster twice in 1999 and 2001. She received prednisolone and methylprednisolone (8 mg, b.i.d, PO) as an immunosuppressive agent for her underlying diseases starting from October 2000.

**Figure 1:**
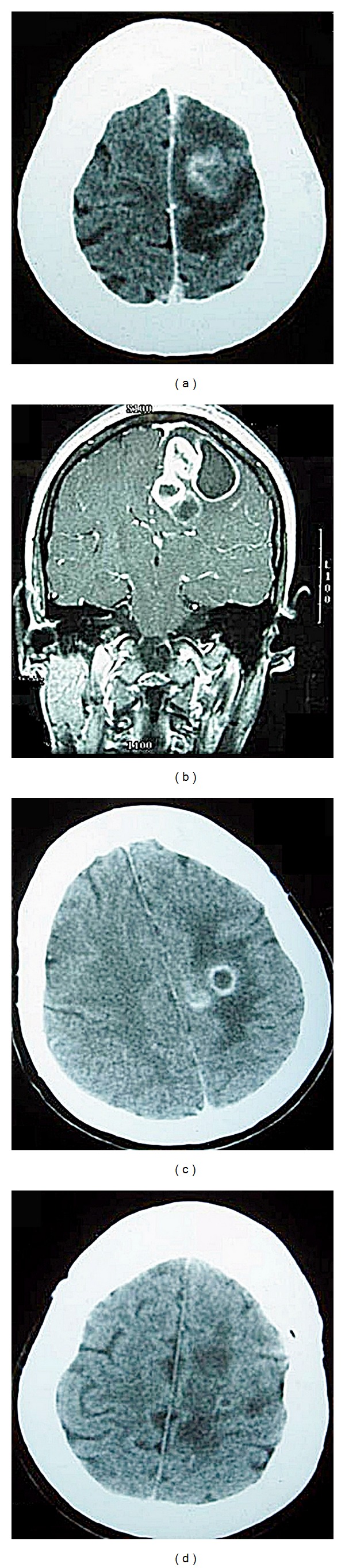
(a) Brain computed tomography (CT) showed ring enhanced nodules with extensive perifocal edema over left frontoparietal region on February 16, 2002. (b) Coronal enhanced T1-weighted magnetic resonance image (MRI) on February 26, 2002, showed multiple conglomerated lesions over left posterior frontal lobe with enhancement. (c) Brain CT on April 8, 2002, and (d) April 15, 2002, showed the gradual regression of the abscesses.

The patient initially suffered chills and fever and antibiotics were given at a local clinic for a respiratory infection for 5 days. Unsteady gait was noted then and she was transferred to our emergency department by the request of her family where pneumonia was diagnosed. Brain computed tomography (CT) at our emergency department on February 16 revealed multiple ring enhanced nodules (20 mm in the largest diameter) with extensive perifocal edema over the left frontoparietal lobe (Figure 1). Stereotaxic biopsy on February 18 determined the presence of gliosis and low grade astrocytoma with inflammation. Empirical antibiotics cefazolin and gentamicin were prescribed. Due to progressive right limbs weakness, dyspnea, loss of light reflex, and drowsy consciousness (M6 to M4) with headache and vomitus, craniotomy was performed without delay on February 28 to remove the abscess. The abscess culture showed* Nocardia* spp. 3 days later.

Sulfamethoxazole (1.2 g, Q6h, iv)/trimethoprim (240 mg, Q6H, iv) was then added according to the culture results. However, progressively reduced muscle power over the left limbs was still found 21 days after the operation. Imipenem/cilastatin (500 mg, q6h, iv) was added according to the suggestion of the infectious disease doctor. During the 4-month course of antibiotic use, the size of the cerebral abscess decreased gradually in the series of follow-up brain CTs on April 8 and April 15 (Figure 1) and the clinical symptoms improved at the same time. She was then discharged from our hospital on June 28. No abscess recurrence was noted and her status was considered to be bed-ridden.

During the hospitalization, an intermittent fever with pneumonia (*Acinetobacter baumannii* and* Pseudomonas aeruginosa*), a perineal candida infection, and a urinary tract infection (*Acinetobacter baumannii*) were also noted, but no evidence of a nocardial species or tuberculosis was found in any of these cultures. Following discharge, rehabilitation programs were arranged at our outpatient department. Her death occurred and her family was informed on December 7, even with attempted resuscitation at our emergency room.

### 2.2. Case 2

A 24-year-old female with systemic lupus erythematosus complicated with anemia, thrombocytopenia, and moderate pericardial effusion received prednisolone (40 mg, qd, PO) as an immunosuppressive agent beginning in December 2005. No other systemic disorders were diagnosed. She was an amanuensis without the habit of drinking or smoking. Long-term moxifloxacin was prescribed for pneumonia in the beginning of 2006.

**Figure 2:**
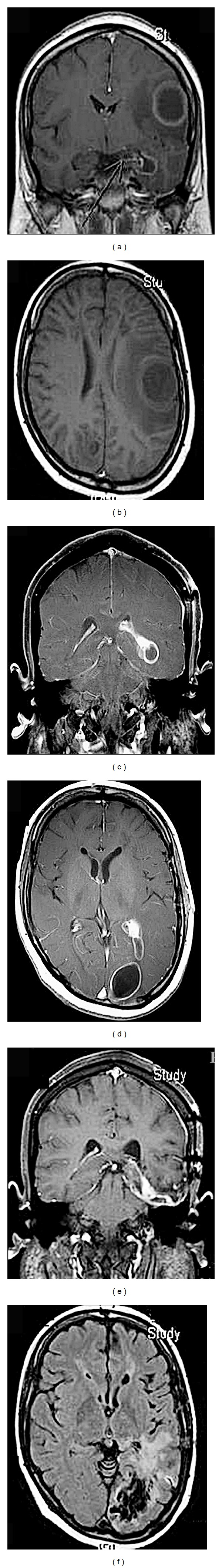
(a) Coronal and (b) axial brain MRI on August 5, 2006, showed the lesion over left temporal region. (c) Coronal and (d) axial brain MRI on August 24, 2006, revealed interval progression of the other abscess in the left occipital regions and the left lateral ventricle. (e) Coronal and (f) axial brain MRI on October 26, 2006, revealed interval regression of the abscesses over the left occipital regions and the left lateral ventricle. Previous residual brain abscesses and ventriculitis subsided.

She then presented at our hospital with a right facial spasm along with a dull pain that radiated to the frontal area. She was afebrile initially, but right side weakness, slurred speech, inappropriate facial muscle power, and leukocytosis (WBC 16200/uL) were noted. A cerebrovascular accident or transient ischemic attack first occurred at the beginning of July 2006. The survey of the brain magnetic resonance imaging (MRI) on August 5 showed multiple rim enhanced lesions over the left temporal and bilateral occipital regions with diffusion restriction and perifocal edema indicative of multiple brain abscesses (35 mm in the largest diameter) (Figure 2). Therefore a small, left craniotomy for aspiration was performed on August 7. The thick capsule walls were preserved and the content yielded 20 mL yellowish-green pus where nocardial growth was cultured. Sulfamethoxazole (1.2 g, q6h)/trimethoprim (240 mg, Q6H) was given according to the laboratory data.

After antibiotic use, the MRI on August 24 (Figure 2) still revealed progression of the abscesses in the bilateral parietal regions and the left lateral ventricle with developing hydrocephalus. Sulfamethoxazole/trimethoprim was changed to meropenem trihydrate (500 mg, q8h) on August 29 and amikacin (450 mg, Q12h) on August 31 in accordance with the suggestions of the infectious disease department. Stereotaxic aspiration with the assistance of CT was arranged on September 1 for the bilateral occipital abscess and the pathology report still revealed* Nocardia* spp. Signs of increased intracranial pressure (IICP) were noticed after the biopsy and an emergency brain CT on September 3 showed left brain swelling with subfalcine and uncal herniations. Therefore, craniotomy guided by sonography was used to remove the hematomas and excise of the left mesial temporal abscesses on September 4. We continued the meropenem and amikacin administration and she was transferred to the rheumatic department ward on September 19 with a projected smooth recovery. Then sulfamethoxazole (800 mg, b.i.d, PO)/trimethoprim (160 mg, b.i.d, PO) was added once again on September 21 and it became the only antibiotic after November 14.

The brain MRI on October 26 (Figure 2) showed that the previous residual brain abscesses and ventriculitis decreased in size. After 5 months of hospitalization with antibiotic control, there was no recurrence of abscesses, but homonymous hemianopia and alexia were noted during follow-up. The patient lost her job due to the above neurological deficits after discharge on January 7, 2007. Sulfamethoxazole/trimethoprim was used until May 29. In 2008, she received right and then left total hip replacement due to avascular necrosis which was considered a complication of long-term steroid use.

### 2.3. Case 3

A 51-year-old male from a rural town had history of bronchial asthma and pneumoconiosis for 4 years. He received prednisolone (10 mg, qd, PO) to control the obstructive lung related asthma starting in August 1999. He initially presented to our institute with dyspnea which was suspected to be an asthma attack in February 2002. Due to respiratory failure, he was intubated with a mechanical ventilator used in our intensive care unit where systemic steroid and a bronchodilator were added to treat the underlying respiratory disease. A fever episode which was considered* E. coli* pneumonia related was noted on February 15, so cefazolin (1gm, q8h, iv) was shifted to cefuroxime (750 mg, q8h, iv), and then amikacin (400 mg, q12h, iv) was added on February 20. Tracheostomy was performed on February 21 due to poor condition and inability to wean the patient off the ventilator. A colonoscopy performed on February 26 to investigate bloody stool revealed pseudomembrane colitis and polyps where adenocarcinoma was identified by biopsy. Cefuroxime and amikacin were discontinued due to no significant pneumonia lesion.

**Figure 3:**
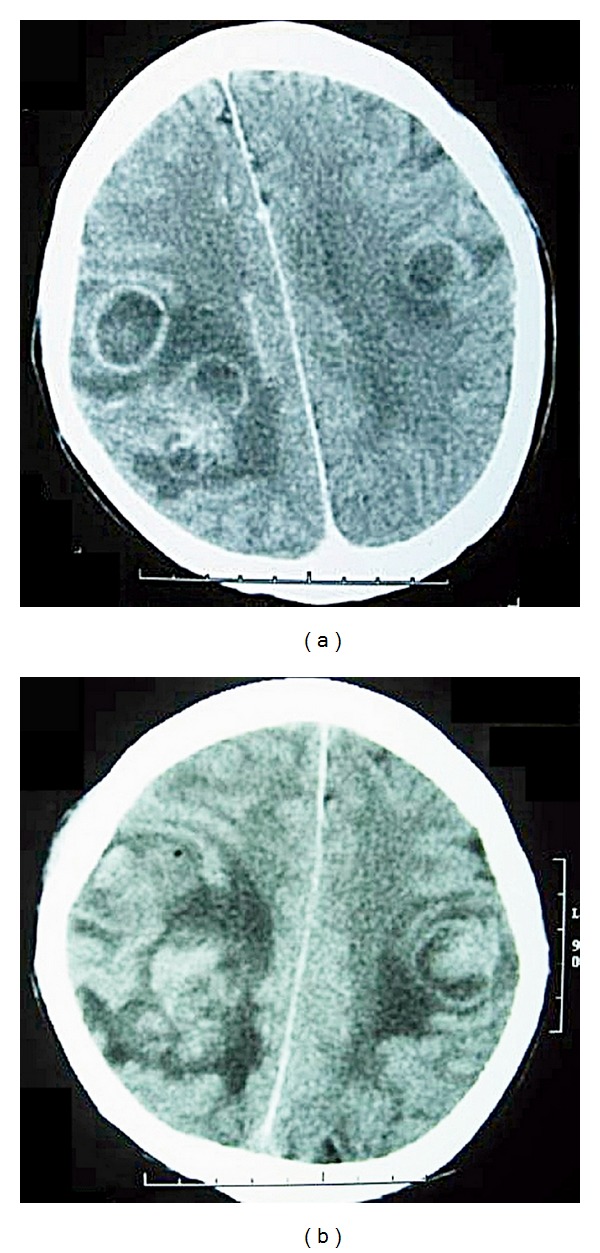
(a) Brain CT with contrast enhanced showed multiple solitary abscesses over right temporal and bilateral frontal, parietal area with brain edema on April 2, 2002. (b) Brain CT on April 9, 2002, found severe brain edema even under the mannitol and dexamethasone use.

In March 2002, steroid myopathy or sedative agent related quadriplegia and seizure attacks were noted. However, muscle weakness that persisted after the adjustment from systemic steroid to oral prednisolone and leukocytosis without fever was noted on March 18. Metronidazole was added for pseudomembranous colitis from to March 15 to March 29. The contrast enhanced brain CT on April 2 revealed multiple solitary brain abscesses over the right temporal and bilateral frontal area, but colon cancer with brain metastasis could not be ruled out (see Figure 3). Due to reoccurrence of seizure attacks on April 4 even with an adequate dilantin level and an IICP sign, stereotaxic aspiration for the right frontal abscesses assisted by CT was performed on April 8. Vancomycin (1 g, q12h, iv) and ceftriaxone (2 gm, q12h, iv) were temporarily used afterwards. Pus culture of the abscesses resulted in* Nocardia* species and sulfamethoxazole (800 mg, q6h, iv)/trimethoprim (160 mg, q6h, iv) was used to replace vancomycin 3 days later, but the clinical condition worsened rapidly. The follow-up brain CT found severe brain edema, even with a mannitol and dexamethasone drip. Tachycardia was noted and complete electrocardiogram showed paroxysmal supraventricular tachycardia (PSVT). We considered the IICP sign to be related to brain herniation and advised craniotomy for him, but his family refused further surgical intervention. Intermittent apnea was found in the afternoon of April 12 and severe brain edema with brain herniation was identified. By the request of the family, the patient was discharged and then died of brainstem failure.

## 3. Discussion

Members of the genus* Nocardia* are gram-positive, bacillary, and branching bacteria who belong to the family Nocardiaceae though the branching rods of the partially acid-fast actinomycete were often misclassified as fungi in the past [3–5]. They are ubiquitous in the environment, living in soil, organic matter, and water [6]. The organism enters the body via inhalation or direct percutaneous inoculation. Nocardial infections often have a hematogenous route of infection from a primary lung focus [7]. If subsequent hematogenous dissemination occurs, it may lead to the infection of any organ, with a particular predilection for the central nervous system (CNS) [6]. Nocardial infections are commonly encountered in patients with immunocompromised states which include human immune deficiency virus (HIV) infection, alcohol abuse, alveolar proteins, diabetes mellitus, neoplasias, and organ transplants [7].

Nocardial brain abscesses are extremely rare; only 3 cases have been documented at our institution over the past 10 years. All three cases were immunocompromised patients undergoing steroid control for more than half a year.* Nocardia asteroides* in the* Nocardia* genus is the most frequent cause of nocardial disease in humans and accounts for more than 80% of invasive infections resulting in systemic and central nervous system disease [1].* N. nova* complex,* N. abscessus*,* N. transvalencesis*,* N. farcinica*,* N. cyriacigeorgica*, and* N. brasiliensis* have become some of the most common isolates among the over 30 species of* Nocardia* with clinical significance [8].

According to the review article of Mamelak et al., coexisting nocardial infections outside the CNS occur in up to 71% of patients [2]. Pulmonary nocardiosis commonly precedes CNS nocardiosis, but no pathological evidence was found that would indicate that a pulmonary or other infection caused by* Nocardia* spp. was associated with the cases in our series which is different from the previously documented literature. All blood cultures were negative for* Nocardia* spp. After tracing their clinical histories, it was discovered that they all had previous systemic infections via respiratory tract and had received long-term antibiotic use. The microenvironment changes may have benefited and lead to the dominance of the* Nocardia* spp. and allowed for the progression to multiple cerebral nocardiosis via the hematic route. Misdiagnosis or mistakes in diagnosis could lead to inadequate antibiotic use that could lead to the elimination of the* Nocardia* spp. in blood and respiratory system but not those in the central nervous system.

The fact that all cases received long-term steroids for their underlying diseases implies that a persistent immunosuppressed condition changes the bionomics of the microorganisms in the body of the Asian race. Multiple pathogenic bacteria contributed to respiratory or urinary tract infections at the same time, but they did not lead to cerebral lesions. Therefore,* Nocardia* spp. is likely to have the ability to penetrate the blood-brain barrier by a specific pathway, especially for Asians, causing multiple brain abscesses. Errors in the antibiotic given initially suppress the growth of the other microbes and offer an environment suited for the spread of the* Nocardia *spp.

### 3.1. Immunocompromised Hosts

*Nocardia* has become a significant opportunistic pathogen in the last two decades due to the recent increase in the number of immunocompromised patients that have occurred with advances in organ transplantation [9–11], severe SLE (like Case 2) [12], systemic therapy for cancer, long-term corticosteroid use [13], and the dramatic emergence of acquired immunodeficiency syndrome (AIDS) [9, 14]. Long-term corticosteroids use, malignancy, and localized pulmonary disease are the most commonly recognized risk factors [15]. The comorbidities of these patients made it more difficult to make the appropriate diagnosis and treatment plan which lead to poor prognosis.

## 4. Diagnosis

Infection of the brain by* Nocardia* species is often insidious in onset and difficult to diagnose and treat successfully. They are often misdiagnosed as malignant brain tumors (like Case 1) [16], vasculitis, or stroke (like Case 2) [2, 4]. A definitive diagnosis may not be possible without collecting a specimen from the lesion. Diagnostic aspirations or biopsies become more important in immunocompromised patients who are more likely to have atypical infections and neoplasms [17]. In our series, multiple lesions and higher mortality rates give rise to diagnostic and therapeutic difficulties. Susceptibility testing and subtyping for the* Nocardia* spp. were not performed for all patients in our institute most likely due to not being arranged as part of a routine schedule, the lack of standardized methods, or the difficulty in growing the organisms uniformly [7]. While nocardial cultures often demonstrate growth too late to be clinically useful or are discarded too early to allow for the growth of* Nocardia*, pyrosequencing (PS), a new semiautomatic molecular genotyping method for nucleic acid sequencing-by-synthesis, may be an efficient means for discriminating microbial species, types, and strains and detecting genetic mutations that confer resistance to antibiotics [18, 19]. Until recently, clinical laboratory testing could not distinguish* N. farcinica* from other members of the* N. asteroides* complex [20]. In our series, all abscesses progressed and showed resistance to initial antibiotic use. We highly suspect* N. farcinica* to be the primary species. PS could be especially helpful in immunocompromised patients and lead to early diagnosis and identification of* N. farcinica* which shows unique resistance patterns to many antibiotics including ciprofloxacin, imipenem, co-trimoxazole, and amikacin and contributes to a higher mortality rate [5, 21].

### 4.1. Treatment

Sulfonamides and trimethoprim/sulfamethoxazole, considered standard medical treatment due to optimal cerebrospinal fluid (CSF) drug levels for nocardial infections, have been in clinical use for half a century [2]. In our three cases, the medical regimens all include it, but it may not be suitable for all cases of nocardiosis. In Case 2, the use of the combination of amikacin and imipenem for the initial blind treatment, especially for the notoriously resistant* N. farcinica* in the early course of treatment, may have improved the clinical outcome [22–24]. Alternatives including linezolid [25], third-generation cephalosporins, including ceftriaxone and cefotaxime [13], and minocycline [26] could be considered.

**Figure 4:**
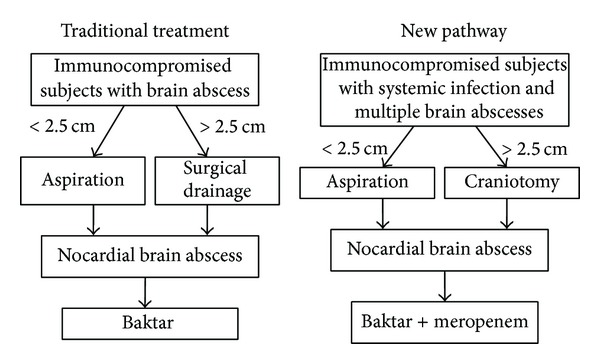


Surgical intervention or medical treatment for nocardial brain abscesses remains inappropriate until culture confirmation [16, 27]. However, to prevent delays in accurate diagnosis and following treatment, aggressive approaches are required, especially for multiple nocardial brain abscesses following systemic infections in immunocompromised subjects. Surgical aspiration of nocardial brain abscesses is indicated for patients diagnosed with an abscess of greater than 2.5 cm traditionally, but aggressive craniotomy must be taken for these hosts with systemic infections and multiple brain lesions [28]. Due to the progression of the abscesses, we advised further craniotomy for Case 2 and Case 3, but it was refused by the family in Case 3. Better recovery was achieved in Case 2 although moderate disability was noted. Time is critical in the treatment of progressive nocardial abscesses and delays in correct treatment have drastic consequences. To prevent excessive neurological deficits and life threatening outcomes, early diagnosis and early neurological drainage by surgery are reasonable for these complex subjects (Figure 4).

It is still unknown whether the discontinuation or the adjustment of immunosuppressive agents, like steroids, changes the outcome of nocardiosis in immunocompromised patients. More review articles and a larger number of cases are needed to test this view and to achieve a balance between nocardial infection and the underlying comorbidities. The appropriate use of immunosuppressive agents will become more important as the number of immunocompromised individuals grows worldwide.

### 4.2. Clinical Outcome

According to the review article written by Mamelak et al. [2], a nocardial brain abscess carries a mortality rate more than three times higher than other bacterial cerebral abscesses.

Mortality rates of 55% and 20% in immunocompromised hosts and immunocompetent patients were reported, respectively. These rates rise to as high as 66% with multiple abscesses [29]. In our series, Case 3 died directly due to the nocardial brain abscess. Case 1 most likely died due to her underlying comorbidities. The only surviving patient, Case 2, still suffered from homonymous hemianopia and alexia which were considered complications from the abscesses. She lost her job due to the above dysfunctions, so nocardial brain abscesses remain a life-threatening disorder that not only wastes abundant financial and social resources but also leads to irreversible neurological deficits. We believe that effective communication between the neurosurgical and microbiological departments is critical in the course of the treatment for these patients.

## 5. Conclusion

Although nocardial brain abscesses constitute only 2% of all brain abscesses, it is much more common cause of brain abscesses in patients who are immunocompromised. Long-term corticosteroid use is a recognized risk factor for the growth of nocardial brain abscesses. We reviewed the patients with nocardial brain abscesses from 2001 to 2011 at our institute and all of them were immunocompromised with long-term steroid use and received multiple antibiotics for pneumonia prior to the formation of multiple cerebral abscesses. According to our tragic experiences, we propose a more aggressive attitude for the treatment of these subjects. Early diagnosis and appropriate antibiotic use should be implemented promptly, and aggressive craniotomy should be performed for multiple nocardial brain abscesses in patients with systemic infections and an immunocompromised status.

## Figures and Tables

**Table 1:** Summary of patient characteristics.

	Case 1	Case 2	Case 3
Age	40	24	51

Gender	F	F	M

Occupation	Housewife	Amanuensis	Worker of pottery

Comorbidities	Systemic sclerosis with Raynaud's syndrome (pulmonary tuberculosis, Herpes zoster, inactive)	Systemic lupus erythematosus	Bronchial asthma, pneumoconiosis, pulmonary tuberculosis, inactive, pseudomembrane colitis, colon adenocarcinoma

Immunosuppression agent	Steroids	Steroids	Steroids

Initial symptoms and signs	Fever; unsteady gait	Right facial spasm with dull pain; slurred speech; afebrile leukocytosis	Fever with leukocytosis; quadriplegia; seizure attacks

Abscess number	Multiple	Multiple	Multiple

Abscess size (in the largest diameter, mm)	10	30	30

Abscess progression	Yes	Yes	Yes

Abscess localization	L, F, P	B, T, O	B, T, F, P

Following treatment	Change to second-line antibiotics	Change to second-line antibiotics; stereotaxic aspiration; craniotomy	Refused craniotomy

3-month outcome	Right hemiplegia	Homonymous hemianopia; alexia	Death

1-year outcome	Death	Homonymous hemianopia; alexia	Death

Other sites involved	Nil	Nil	Nil

^ a^Duration from symptoms onset to diagnosis (day)	14	7	38

Antibiotics for abscesses	Oxacillin; ceftriaxone; imipenem; sulfamethoxazole/trimethoprim	Sulfamethoxazole/trimethoprim; meropenem trihydrate; amikacin	Sulfamethoxazole/trimethoprim

Duration of antibiotics use (day)	75	294	4

Surgical intervention	Craniotomy with guided sonography for the aspiration of the abscesses	Craniotomy with guided sonography for the aspiration of the abscesses	Stereotaxic aspiration

^a^Time from onset of neurological symptoms to laboratory proven results.

M: male; F: female; L: left; B: bilateral; F: frontal; P: parietal; T: temporal; O: occipital.
